# Distinct resting-state functional connectivity patterns of Anterior Insula affected by smoking in mild cognitive impairment

**DOI:** 10.1007/s11682-023-00766-6

**Published:** 2023-05-27

**Authors:** Tianyi Zhang, Qingze Zeng, Kaicheng Li, Xiaocao Liu, Yanv Fu, Tiantian Qiu, Peiyu Huang, Xiao Luo, Zhirong Liu, Guoping Peng, for the Alzheimer’s Disease Neuroimaging Initiative (ADNI)

**Affiliations:** 1 Department of Neurology, The 1st Affiliated Hospital of Zhejiang University School of Medicine, No.79 Qing-Chun Road, Shang- Cheng District, Hangzhou, 310002 China; 2 Department of Radiology, The 2nd Affiliated Hospital of Zhejiang University School of Medicine, Hangzhou, China; 3 Department of Neurology, The 2nd Affiliated Hospital of Zhejiang University School of Medicine, Hangzhou, China; 4 Department of Radiology, Linyi People’s Hospital, Linyi, China

**Keywords:** Mild cognitive impairment, Resting-state functional MRI, Functional connectivity, Insula, Smoking

## Abstract

Smoking is a modifiable risk factor for Alzheimer’s disease (AD). The insula plays a vital role in both smoking and cognition. However, the smoking effects on insula-related networks in cognitively normal controls (CN) and mild cognitive impairment (MCI) patients remain unknown. We identified 129 CN (85 non-smokers and 44 smokers) and 83 MCI (54 non-smokers and 29 smokers). Each underwent neuropsychological assessment and MRI (structural and resting-state functional). Seed-based functional analyses in the anterior and posterior insula were performed to calculate the functional connectivity (FC) with voxels in the whole brain. Mixed-effect analyses were performed to explore the interactive effects on smoking and cognitive status. Associations between FC and neuropsychological scales were assessed. Mixed-effect analyses revealed the FC differences between the right anterior insula (RAI) with the left middle temporal gyrus (LMTG) and that with the right inferior parietal lobule (RIPL) (*p* < 0.01, cluster level < 0.05, two-tailed, gaussian random field correction). The FC of RAI in both LMTG and RIPL sees a significant decrease in MCI smokers (*p* < 0.01). Smoking affects insula FC differently between MCI and CN, and could decrease the insula FC in MCI patients. Our study provides evidence of neural mechanisms between smoking and AD.

**Supplementary Information:**

The online version contains supplementary material available at 10.1007/s11682-023-00766-6.

## Introduction

Alzheimer’s disease (AD) is a common neurodegenerative disease characterized by progressive cognitive impairment. Although there is no effective medicine to prevent the onset of AD or reverse the disease progression, the reduction of risk factors could slow down the disease progression. Smoking is one of the modifiable risk factors for dementia. Smokers are approximately 1.6 times more likely to suffer from AD than non-smokers (Reitz et al., 2007). Acute cognitive effects of nicotine, one of the main components of tobacco, were characterized by enhanced selective attention and memory (Swan & Lessov-Schlaggar, 2007; Valentine & Sofuoglu, 2018). However, compared with non-smokers, active smokers had poorer performance in global cognitive function and multiple neurocognitive domains (Durazzo et al., 2012; Ott et al., 2004; Swan & Lessov-Schlaggar, 2007). The effects of smoking on brain function at different cognitive stages remain unknown.

The nicotinic acetylcholine receptor (nAChR) is a potential target for nicotine, and it has the highest density in the insula (Picard et al., 2013). The insula plays a pivotal role in smoking craving and regulating withdrawal during abstinence (Abdolahi et al., 2015; Morales et al., 2014). Naqvi et al. reported that the lesions to the insula disrupt smoking addiction, which updated our understanding of smoking-related structures in the brain (Naqvi et al., 2007). Among current smokers, patients with damage to the insula suffered fewer and less severe withdrawal symptoms than those without insula injury (Abdolahi, Williams, Benesch, Wang, Spitzer, Scott, Block and van Wijngaarden, 2015). A voxel-based morphometry study showed that cigarette smoking was associated with atrophies in many regions, including the insula (Fritz et al., 2014). One functional MRI (fMRI) study demonstrated that heavy smokers exhibited increased regional homogeneity in the posterior cingulate cortex and insula (Yu et al., 2013). Increased functional connectivity (FC) of the anterior insula (AI) improved smoking cessation outcomes (Wang et al., 2020a).

Structurally, the insula is divided into anterior and posterior lobules by the central insular sulcus (Benarroch, 2019). AI is involved in self-awareness, emotional responses, and cognitive control. At the same time, the posterior insula (PI) is functionally connected with areas involved in sensorimotor-interoceptive functions (Benarroch, 2019). The insula, especially the anterior lobe, plays a key role in high-level cognitive control and attentional processes (Menon & Uddin, 2010). Insula atrophy is related to cognitive impairment in neurodegenerative diseases such as AD, Parkinson’s disease, and frontotemporal dementia (Bejanin et al., 2020; Li et al., 2016; Song et al., 2011). The insula is a key hub of cognition-related functional brain networks (Molnar-Szakacs & Uddin, 2022). Neuroimaging studies showed a wide array of functional and structural connectivity between the insula and the frontal, temporal, parietal, occipital lobes as well as limbic regions (Benarroch, 2019; Ghaziri et al., 2017). Connection patterns are different between the anterior and posterior insula (Cauda et al., 2011; Cloutman et al., 2012). Resting-state functional connectivity (rsFC) can is commonly measured by the correlation between different brain regions. Since the insula acts as the mutual node of both smoking and cognition, insula FC could be the potential imaging marker to account for the different effects of smoking in different cognitive stages.

We investigated the effects of smoking on insula-related brain networks in 129 cognitively normal (CN) controls and 83 MCI patients using seed-based FC analyses, which may provide the potential neural mechanism of smoking effects on MCI. Based on previous studies (Liu et al., 2018; Stoeckel et al., 2016; Wang et al., 2006), we hypothesized that smoking might increase the insula FC in CN controls but decrease it in MCI patients.

## Materials and methods

### Participants

All data used in this study were from Alzheimer’s Disease Neuroimaging Initiative (ADNI) database (http://adni.loni.usc.edu/). This ongoing project was launched in 2003 to develop clinical, neuropsychological, and neuroimaging biomarkers for early disease detection and progression monitoring of AD. Informed written consent was obtained from all participants at each site. This study was approved by the Institutional Review Boards of all of the participating institutions.

In this study, we included 378 CN subjects and 182 MCI patients who have completed resting-state functional MRI from ADNI 3 database before December 21st, 2020. Criteria for CN controls were defined as: (1) Mini-Mental State Examination (MMSE) between 24 and 30 (inclusive); (2) clinical dementia rating (CDR) score of 0; (3) no signs of depression (Geriatric Depression Scale, GDS < 6) or dementia. We defined MCI as: (1) MMSE between 24 and 30 (inclusive); (2) CDR score of 0.5; (3) a memory complaint; (4) objective memory loss defined as scoring below an education-adjusted cut-off score on delayed recall of the Wechsler Memory Scale logical memory test; (5) general cognition preserved at the time of screening; (6) no signs of depression. We excluded participants with a history of obvious head trauma, alcohol/drug abuse, other neurological or significant psychiatric disorder, or a significant vascular disease risk history defined as Hachinski Ischemia Scale (HIS) > 4. After that, we classified CN controls and MCI patients into smoking and non-smoking groups, respectively, based on self-report smoking history.

We identified 378 right-handed cognitively intact healthy participants (including 333 non-smoking CN and 45 smoking CN) and 182 MCI patients (including 149 non-smoking MCI and 33 smoking MCI) with T1 weighted structural scan, resting-state fMRI scan, and comprehensive neuropsychological assessments. The details of the flow chart could be seen in Supplementary Material 1, Figure S1.

### Neuropsychological assessments

Each subject finished a neuropsychological examination to assess the general mental status and other cognitive domains, including visuospatial function (Clock-Drawing Test, CDT), memory (Auditory Verbal Learning Test, AVLT; Immediate Story Retell, IST; Delayed Story Retell, DST), language (Semantic Verbal Fluency, SVF), processing speed (Trail-Making Test, Part A, TMT-A), and executive function (Trail-Making Test, Part B, TMT-B).

### Data acquisition

We obtained structural images using a T1 weighted sequence with the following parameters: echo time (TE) = 2.98 ms; repetition time (TR) = 2300 ms; 170 sagittal slices; within plane FOV = 256 × 240 mm^2^; voxel size = 1 × 1 × 1 mm^3^; flip angle = 9°; bandwidth = 240 Hz/pix. The resting-state fMRI data were obtained by using an echo-planar imaging (EPI) sequence with the following parameters: 197 time points; TR/TE = 3,000/30 ms; slice number = 48; slice thickness = 3.3 mm; matrix = 64 × 64. The detailed MRI parameters were shown in http://adni.loni.usc.edu/methods/documents/.

### Imaging pre-processing

Data pre-processing were performed by the Data Processing Assistant for Resting-state fMRI (DPARSF, Yan and Zang; http://rfmri.org/DPASFA) based on Statistical Parametric Mapping 12. The first 10 volumes of the resting-state fMRI images were removed due to the signal equilibrium and the subject’s adaptation to the scanning noise. The remaining images were corrected for timing differences between slices and head motion. We discarded image data with more than 3 mm maximum displacement in any x, y, or z directions or 3° of any angular motion. 49 subjects were excluded because of head motion (including 29 non-smoking CN, one smoking CN, and 19 non-smoking MCI). Finally, we identified 304 non-smoking CN, 44 smoking CN, 130 non-smoking MCI, and 33 smoking MCI (Supplementary Material 1, Figure S1). After the subjects being identified, the fMRI images were normalized to the EPI template, resampled into 3 × 3 × 3 mm^3^, and smoothed with a Gaussian kernel of 6 × 6 × 6 mm^3^ full widths at half maximum. We conducted covariates regression to control the residual effects of motion and other non-neuronal factors, including 24 head motion parameters and other parameters of white matter, cerebrospinal fluid, and mean global signal. In the end, filtering (0.01–0.08 Hz) was performed to remove bias from high-frequency physiological noise and low-frequency drift.

### Propensity score matching

We used estimated propensity scores to match patients with non-smoking and smoking subgroups in CN and MCI to reduce the effect of selection bias. Each patient’s propensity score was estimated using multiple logistic regression analysis based on age, sex, and years of education covariates. A 2: 1 matching was used to pair non-smoking participants with smoking CN and MCI. Significant testing and standardized difference (d) were applied to assess the balance of covariates before and after propensity score matching (PSM) (Supplementary Material 2, Table S1). The PSM was performed with SPSS 26, and 85 non-smoking CN, 44 smoking CN, 54 non-smoking MCI, and 29 smoking MCI were selected from the initial population (Supplementary Material 1, Figure S1). All the results in this study were based on the PSM population, except those specifically indicated.

### Functional connectivity analysis

**Fig. 1:**
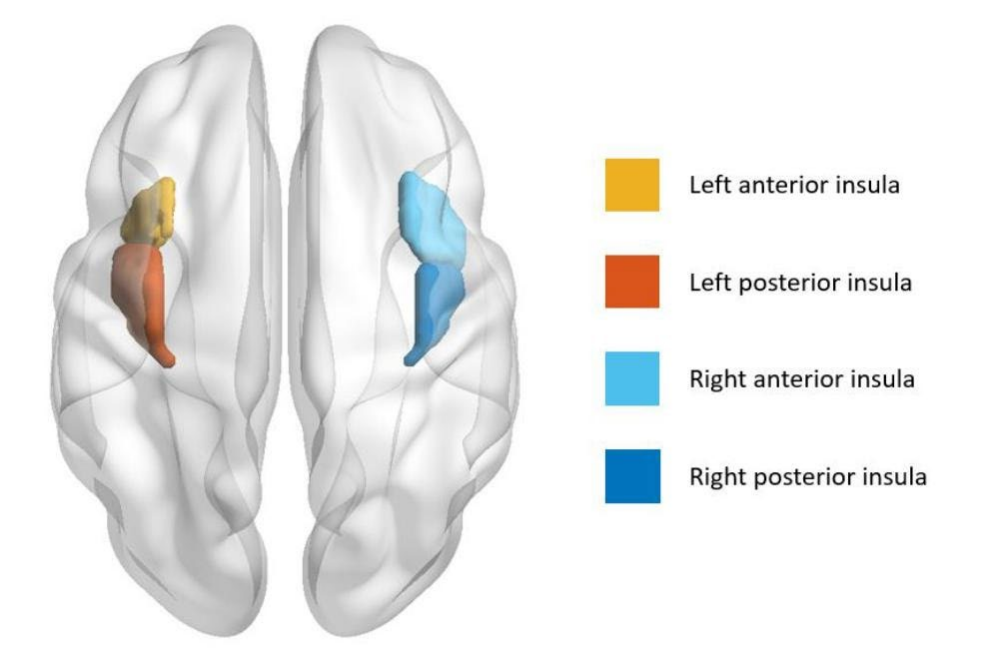
Schematic of the left and right insula parcellation

Both left and right insulas were segmented into anterior and posterior subregions to investigate the lateralization and specificity of insular subregions in the smoking effect (Fig. 1), using the analytic scripts downloaded via http://fcon_1000.projects.nitrc.org as seeds (Kelly et al., 2012). The left and right insular subregions masks were resampled to the dimension of our normalized functional image with 3 × 3 × 3 voxel size, then the left and right insular subregions were used as seed regions to extract insular subregions signals from the fractional amplitude of low-frequency fluctuations maps.

We obtained individual seed-to-voxel FC maps using the Dynamic brain connectome (DynamicBC) toolbox (http://restfmri.net/forum/DynamicBC). The seed-to-voxel FC maps were generated by calculating the Pearson correlation between the time course of the seed and whole-brain voxels. After that, the result maps were transformed into zFC maps using Fisher’s Z transformation.

### Statistical analysis

The statistical analyses were conducted by SPSS 26. Results with *p* < 0.05 were considered statistically significant. We described continuous and categorical variables as mean ± standard deviation and percentage, respectively. We used analyses of variance (ANOVA) for age, education, and neuropsychological scores among four groups (degree of freedom is 211). Subsequently, a *post-hoc* two-sample T-test was conducted (*p* < 0.05, Bonferroni corrected). We used the Chi-square test for sex distribution.

The statistical analyses of the FC maps were performed using the DPABI toolbox (Yan et al., 2016). Specifically, we performed a 2 × 2 mixed effect analysis to explore the main effects of condition (smoking status, non-smoking vs. smoking), group (cognitive status, CN vs. MCI) and the interactive effects of smoking × cognitive status. We used age, sex, years of education, and head motion (FD value) as covariates. To avoid the influence of cortical atrophy, we further included grey matter as a covariate. We set the threshold as the voxel level at *p* < 0.01 and the cluster level at *p* < 0.05 after Gaussian random field (GRF) correction. Then, we extracted the FC values from the regions-of-interest with statistically significant differences and further performed *post-hoc* analysis (*p* < 0.01, Bonferroni corrected). Finally, we performed a partial correlation analysis to investigate the correlation between the mean FC values of interactive regions and neuropsychological scores with age, sex, and years of education as covariates (*p* < 0.05, Bonferroni corrected).

## Results

### Demographics

There was no significant difference in sex, age, and education among the non-smoking CN, smoking CN, non-smoking MCI, and smoking MCI (*p* > 0.05) (Table 1). The general cognitive deficits measured by the MMSE showed significant differences among the four groups (*p* < 0.001). In the detailed cognitive domain assessment, there are significant differences among the four groups in memory [IST (*p* < 0.001), DST (*p* < 0.001) and AVLT (*p* < 0.001)], language function [SVF (*p* = 0.02)], attention [TMT-A (*p* < 0.001)] and executive function [TMT-B (*p* < 0.001)]. There is no significant difference in visuospatial function [CDT (*p* = 0.127)]. The *post-hoc* analysis further showed that the two CN groups’ memory, language, and executive functions were significantly better than those in the two MCI groups (*p* < 0.05). In addition, there was no significant difference in cognitive function between the smokers and non-smokers, two CN groups, and two MCI groups.

**Table 1:** Demographic characteristics and neuropsychological scales of the study population in the PSM population

	Non-smoking CN (n = 85)	Smoking CN (n = 44)	Non-smoking MCI (n = 54)	Smoking MCI (n = 29)	F/χ^2^ Value	*p-*Value
Age	75.42 ± 7.33	75.83 ± 7.64	75.76 ± 7.48	76.19 ± 6.80	0.09	0.97
Education	16.33 ± 2.53	16.27 ± 2.61	16.35 ± 2.32	16.24 ± 1.70	0.18	1.00
Sex (F/M)	45/40	23/21	31/23	17/12	0.02	0.91
MMSE	28.98 ± 1.21	29.25 ± 0.81	28.17 ± 1.65	27.52 ± 1.77	2.03	< 0.001 ^abcd^
**Visuospatial**						
CDT	4.69 ± 0.62	4.59 ± 0.62	4.41 ± 0.86	4.45 ± 0.99	2.72	0.127
**Memory**						
IST	14.27 ± 3.76	15.30 ± 3.30	10.89 ± 4.48	11.66 ± 4.86	13.52	< 0.001 ^abcd^
DST	13.25 ± 4.02	14.30 ± 3.56	8.83 ± 4.78	9.69 ± 4.65	15.69	< 0.001 ^abcd^
AVLT	45.45 ± 9.56	46.00 ± 10.30	34.98 ± 8.76	37.86 ± 11.49	15.68	< 0.001 ^abcd^
**Language**						
SVF	20.92 ± 4.85	21.25 ± 5.84	18.93 ± 5.03	18.24 ± 5.42	3.90	0.02
**Processing speed**						
TMT-A	32.31 ± 9.30	29.52 ± 6.78	36.30 ± 12.03	38.76 ± 13.38	2.84	< 0.001 ^bcd^
**Execution**						
TMT-B	79.79 ± 36.12	69.68 ± 27.43	94.69 ± 39.94	103.93 ± 54.88	6.50	< 0.001 ^bcd^

Data are presented as mean ± standard deviation. Abbreviation: PSM: propensity score matching, CN: cognitively normal, MCI: mild cognitive impairment, F: female, M: male, MMSE: Mini-Mental State Examination, CDT: clock drawing test, IST: immediate story retell, DST: delayed story retell, AVLT: auditory verbal learning test, SVF: semantic verbal fluency, TMT-A: Trail-Making Test, Part A, TMT-B: Trail-Making Test, Part B

^a−d^ Post hoc paired comparison further revealed the source of analysis of variance difference respectively (*p* < 0.05, after Bonferroni corrected). ^a^ non-smoking CN vs. non-smoking MCI, ^b^ non-smoking CN vs. smoking MCI, ^c^ smoking CN vs. non-smoking MCI, ^d^ smoking CN vs. smoking MCI.

### Smoking alters the FC of the Insula in MCI

**Fig. 2:**
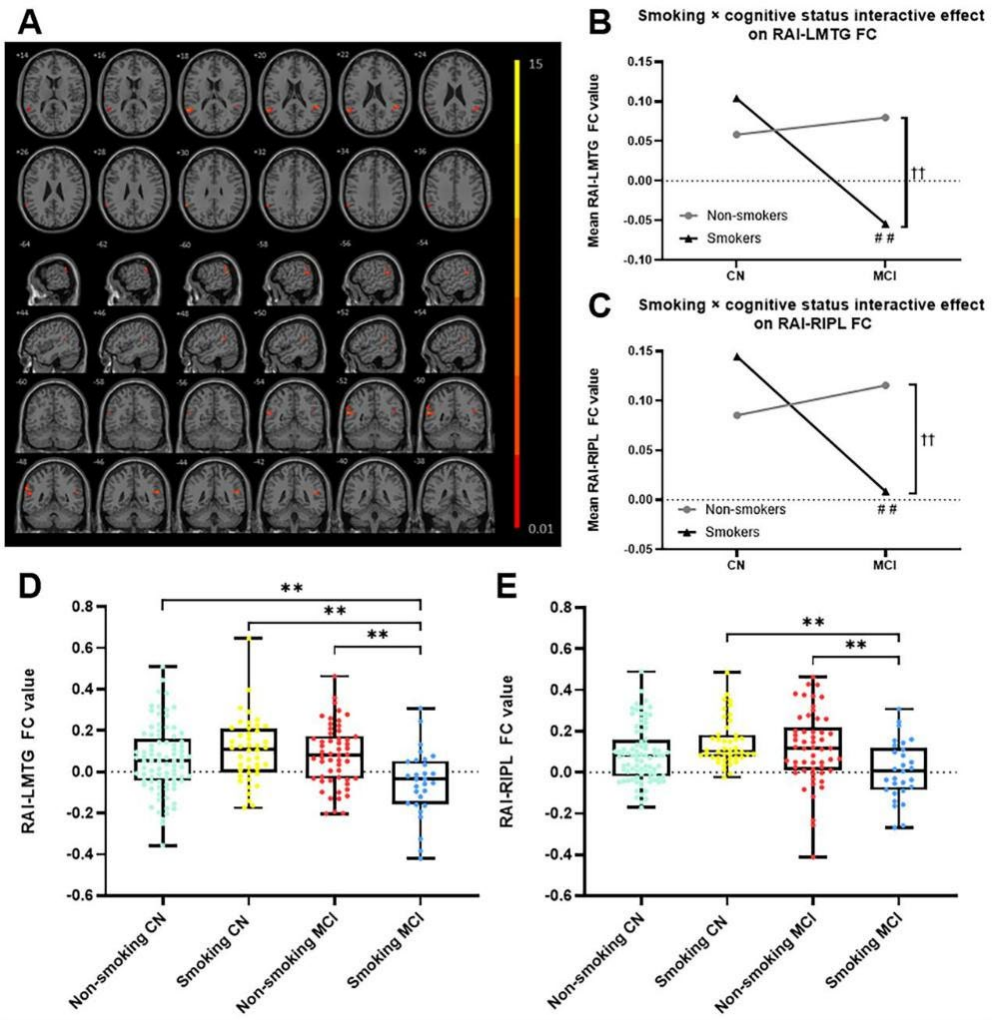
**The significant interactive effects (smoking × cognitive state) identified between the RAI with LMTG and RIPL among non-smoking CN, smoking CN, non-smoking MCI, and smoking MCI in the PSM population** The figure A shows the difference in the RAI FC after adjustments for age, sex, education, and gray matter volume among non-smoking CN, smoking CN, non-smoking MCI, and smoking MCI (GRF corrected, *p* < 0.01 at height and *p* < 0.05 at cluster level). Figures B-E show the *post-hoc* t-test results of RAI-LMTG FC and RAI-RIPL FC values (*p* < 0.01, Bonferroni corrected) **Abbreviation**: RAI: right anterior insula, FC: functional connectivity, LMTG: left middle temporal gyrus, RIPL: right inferior parietal lobule, CN: cognitively normal, MCI: mild cognitive impairment, PSM: propensity score matching, GRF: Gaussian random field. †† *p* < 0.01 between non-smokers and smokers in MCI after Bonferroni correction. ## *p* < 0.01 between CN and smokers after Bonferroni correction. ** *p* < 0.01, two-sample t-test, Bonferroni correction.

**Fig. 3:**
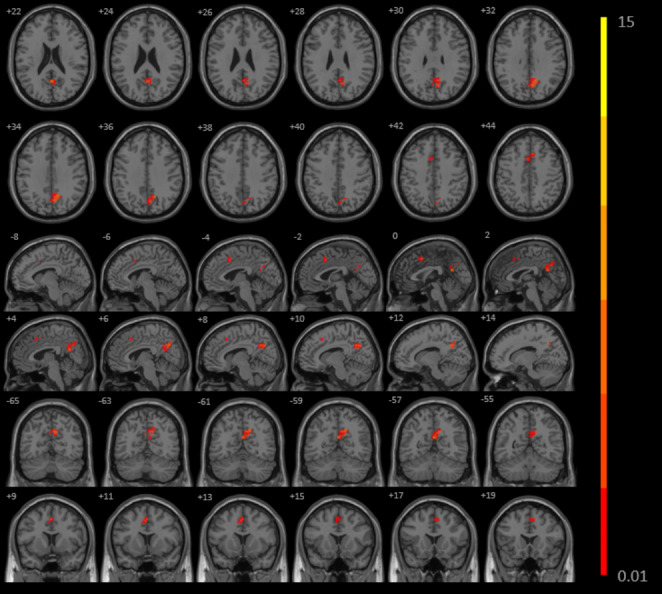
**The significant interaction effects (smoking × cognitive state) identified between the LPI with LP and LCG among non-smoking CN, smoking CN, non-smoking MCI and smoking MCI in the PSM population** The figure shows the difference in the LPI FC after adjustment for age, sex, education, and gray matter volume in non-smoking CN, smoking CN, non-smoking MCI and smoking MCI after-PSM subjects. The four groups have significant interaction between left PI and left precuneus and cingulate gyrus (GRF corrected, *p* < 0.01 at height and *p* < 0.05 at cluster level) **Abbreviation**: LPI: left posterior insula, LP: left precuneus, LCG: left cingulate gyrus, CN: cognitively normal, MCI: mild cognitive impairment, PSM: propensity score matching, GRF: Gaussian random field.

Based on the mixed-effects analysis in non-smoking CN, smoking CN, non-smoking MCI, and smoking MCI, we identified regions where the FC of the insula were significantly altered (corrected by age, sex, education, and gray matter volume): (1) between the right AI and left middle temporal gyrus (RAI-LMTG) (cluster size 48 voxels, *p* < 0.01, cluster level < 0.05, GRF corrected) (Fig. 2A; Table 2); (2) between the right AI and right inferior parietal lobule (RAI-RIPL) (cluster size 25 voxels, *p* < 0.01, cluster level < 0.05, GRF corrected) (Fig. 2A; Table 2); (3) between the left PI and left precuneus (cluster size 102 voxels, *p* < 0.01, cluster level < 0.05, GRF corrected) (Fig. 3; Table 2); (4) between the left PI and left cingulate gyrus (cluster size 37 voxels, *p* < 0.01, cluster level < 0.05, GRF corrected) (Fig. 3; Table 2).

**Table 2:** Brain areas with significant insula FC difference among non-smoking CN, smoking CN, non-smoking MCI, and smoking MCI in the PSM population

Brain region	Cluster size	Peak MNI coordinates			peak-value
		X	Y	Z	
Right anterior insula					
left middle temporal gyrus	48	-60	-51	18	15.28
right inferior parietal lobule	25	48	-42	21	14.45
Right posterior insula					
None					
Left anterior insula					
None					
Left posterior insula					
left precuneus	102	0	-57	21	15.82
left cingulate gyrus	37	-3	12	45	12.94

Abbreviation: FC: functional connectivity, CN: cognitively normal, MCI: mild cognitive impairment, PSM: propensity score matching, MNI: Montreal Neurological Institute, X Y Z coordinates the primary peak locations in the MNI space

To verify the repeatability the results on the basis of the PSM population, we also re-assessed the FC of the insula throughout the whole participants (n = 511) (**Supplementary Material 4, Table S2, Table S3, Figure S3 and Figure S4**). The result indicated that only the interactive effects (smoking × cognitive state) in the right AI FC with LMTG and RIPL remained unchanged (*p* < 0.01, cluster level < 0.05, GRF corrected) (Fig. 2A; Table 2). In addition, we further performed a *post-hoc* region-of-interest analysis for the four areas to make the interactive effect (smoking × cognitive state) clear (Fig. 2B and C ). Specifically, *post-hoc* results showed that RAI FC with LMTG in the non-smoking CN, smoking CN, and non-smoking MCI were significantly higher than in the smoking MCI (*p* < 0.01, Bonferroni corrected). RAI FC with RIPL in the smoking CN and non-smoking MCI were significantly higher than in the smoking MCI (*p* < 0.01, Bonferroni corrected) **(**Fig. 2D and E**)**. No condition (smoking status) effects or group (cognitive status) effects were observed.

### Correlation between the FC of RAI and Neuropsychological Scores

Correlation analysis was further performed between FC and neuropsychological scales. After correction of age, sex and education, no significant associations were found in the overall PSM population. Specifically, partial correlation results across subgroups showed in **Supplementary Material 5, Table S4, Figure S5.**

## Discussion

For the first time, we used resting-state fMRI to compare insula FC changes among the CN and MCI patients with or without a smoking history. We aimed to explore the potential effects of smoking on insula FC in MCI patients. We found that smoking may decrease the insula FC in MCI, which is different from CN. Among smokers, RAI-LMTG FC and RAI-RIPL FC were lower in the MCI patients than in CN. Our results showed that smoking might exert a negative effect on the insula FC in MCI patients. Among CN, however, this effect might be quite different or even the opposite.

Our﻿ study identified significant interactive effects in the right AI FC with the LMTG and RIPL in all participants post-PSM. MTG and IPL correlate with the default mode network (DMN) and the frontoparietal network (FPN) (Igelstrom & Graziano, 2017; Xu et al., 2019). MTG is mainly involved in language, emotion, social cognition, and novelty detection (Papeo et al., 2019; Ren et al., 2020; Wang et al., 2020b). Consistently, one fMRI work showed that MCI patients exhibited increased activity in the MTG compared to CN controls, which might compensate for the loss of function (Qi et al., 2010). Subjects with amyloid deposition showed a more significant volume reduction in the MTG (Hyung et al., 2021). Furthermore, the progressive MCI patients had more lateral temporal lobe (including MTG) atrophy than stable MCI patients (Karas et al., 2008). One study found that patients with subjective cognitive decline were significantly hypometabolic in the MTG (Dong et al., 2021). IPL is an essential part of the DMN. IPL is involved in many cognitive thresholds, such as visuospatial function, reading competency, sensory-motor adaptation, and executive control (Bedard & Sanes, 2014; Crottaz-Herbette et al., 2014; Grabski et al., 2012; Turner & Spreng, 2012; Wang et al., 2012). Compared to CN, progressive MCI patients showed significantly increased IPL connectivity (Esposito et al., 2013). A PET study found that MCI patients showed hypometabolism in several regions, including IPL (Nobili et al., 2010). Previous PET studies indicated that tau and amyloid pathology presumably occurred in IPL in the early stages of AD (Cho et al., 2016; Mattsson et al., 2019). The compensatory hypothesis that the increased brain activity may effectively serve a compensatory role, was thought to occur in AD processes (Bookheimer et al., 2000). This conclusion is consistent with our results that the MCI patients showed relatively higher FC than that of CN among the non-smokers. Increased FC may indicate an increased compensatory brain activity at the cognitive normal stage. Moreover, our results indicate that smoking tends to increase the FC in both MTG and IPL among CN. We hypothesized that smoking might play a protective role when there is no cognitive impairment. There are several explanations. Meta-analysis revealed increased activation of the MTG in response to smoking-related cues in smokers than that to neutral cues (Engelmann et al., 2012). fMRI analysis showed that smokers displayed increased activation in several regions, including RIPL (Chen et al., 2018). Nicotine, the main component of tobacco, was beneficial to working memory function, learning, and attention (Levin et al., 2006).

Our study found that compared to CN, the RAI FC with both LMTG and RIPL decreased in smokers but increased in non-smokers among the MCI patients. Nicotine is a cholinergic agonist for nicotinic acetylcholine receptors (nAChRs) (Benowitz, 2009). Stimulation of central nAChRs results in the release of various neurotransmitters in brain (Benowitz, 2009). Nicotine is not a substrate for acetylcholinesterase. Chronic nicotine consumption may cause prolonged activation of excitatory nAChRs (Penton & Lester, 2009). A previous study indicated that prolonged activation of the nAChR leads to desensitization and upregulation of nAChR density (Zoli et al., 2018). These pathological processes result in an inactive receptor that does not allow ions’ passage (Zoli et al., 2018). When the disease progresses to the stage of cognitive impairment, we speculate that AChR may upregulate to reach saturation and the function is desensitized, resulting in decreased FC. In addition, insular function contributes to the maintenance of smoking behaviors. A previous study demonstrated that the right dorsal AI might be an integral node for integrating information within and across cognitive, affective, and sensorimotor tasks (Uddin et al., 2014). The AI also plays a critical position between the DMN and the central executive network (Menon & Uddin, 2010). There is a limit for the insula to process cognitive tasks: AI is associated with the total capacity or upper functioning limit of cognitive control (Wu et al., 2019). Among smokers, the insula may be loaded with processes of smoking that prevent it from executing the cognitive tasks (Regner et al., 2019). Our results suggested that brain function was less compensated in smokers when cognitive impairment manifested.

### **Limitations**

Our study also had several limitations. Firstly, since the ADNI database is not specifically designed to study the effects of smoking on cognitive impairment, detailed smoking data, such as Fagerström Test for Nicotine Dependence, was insufficient. Future studies with accurate smoking data are needed to verify our work. The quantitative analysis should explore the impact of smoking amount and time on the brain FC of MCI. Secondly, because of the lack of neuropathological data such as cerebrospinal fluid or PET, our diagnosis of cognitive status was only based on clinical symptoms rather than the Amyloid/Tau/Neurodegeneration framework, which may cause some subjects to be mis-grouped. Thirdly, the method of insula segmentation in our study is relatively rough, and more fine-grained method of insula segmentation may lead to different results. Finally, as a preliminary exploration, our study only explored the smoking effects on the insula FC in MCI from a cross-sectional sample. Thus, longitudinal studies are needed to determine the long-term effects of smoking on brain FC during the progression of MCI.

## **Conclusions**

Our study explored the effects of smoking on insula FC on different cognitive stages. We found that smoking significantly affected the AI FC with LMTG and RIPL. AI FC increased in the non-smoking MCI but decreased in the smoking MCI. The lower connectivity strength in RAI-RIPL FC correlated with poor memory and execution function among smokers. The results of our study suggested that brain function was less compensated in smokers when cognitive impairment manifested.

## Electronic supplementary material

Below is the link to the electronic supplementary material.

Supplementary Material 1

## Data Availability

The datasets generated and/or analysed in our study are available in the ADNI database (adni.loni.usc.edu).
