# Supplementary material for: Distinct resting-state functional connectivity patterns of Anterior Insula affected by smoking in mild cognitive impairment
Source: Brain Imaging Behav. 2023 May 27;17(4):386–94. doi: 10.1007/s11682-023-00766-6 (PMC10435406; doi:10.1007/s11682-023-00766-6)
Supplement: Supplementary file 1 — Supplementary Material 1 [file 11682_2023_766_MOESM1_ESM.docx]

## Supplementary Material 1


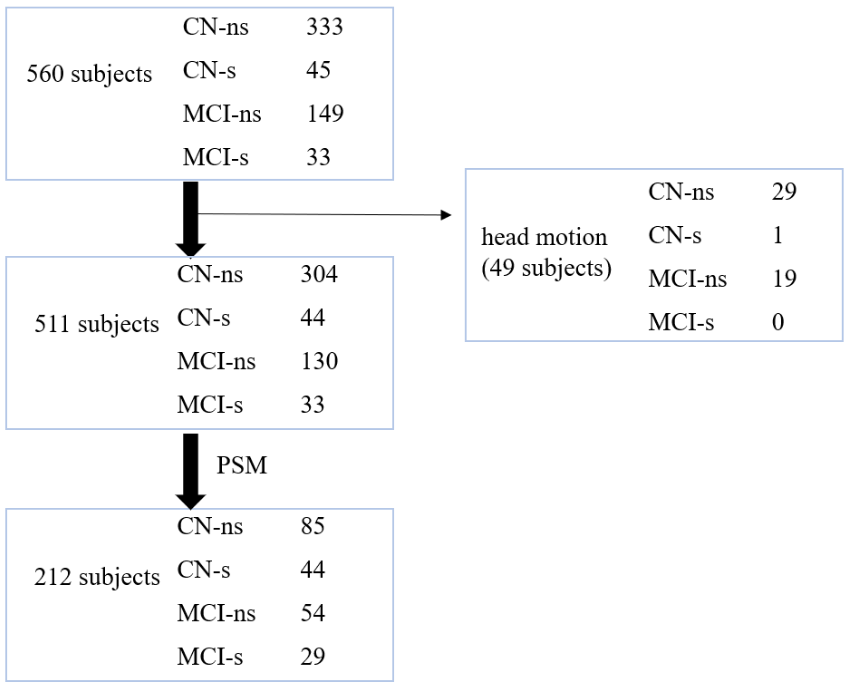


**Figure S1. The screening processes of smoking subjects from the ADNI database before December 21st, 2020.**

We excluded 49 subjects due to head motion. Finally, 85 non-smoking CN, 44 smoking CN, 54 non-smoking MCI, and 29 smoking MCI entered subsequent analyses.

Abbreviations: CN: cognitively normal, MCI: mild cognitive impairment, ns: non-smoking, s: smoking, PSM: propensity score matching

## Supplementary Material 2

**Table S1. Standardized difference (d) before and after PSM in CN and MCI**

|  | Standardized difference (d) in CN | |  | Standardized difference (d) in MCI | |
| --- | --- | --- | --- | --- | --- |
|  | Before PSM | After PSM |  | Before PSM | After PSM |
| Age | 0.374 | -0.039 |  | 0.302 | 0.103 |
| Male | 0.247 | -0.157 |  | 0.137 | -0.033 |
| Education | -0.231 | -0.139 |  | -0.384 | -0.061 |

Abbreviations: PSM: propensity score matching, CN: cognitively normal, MCI: mild cognitive impairment

## Supplementary Material 3


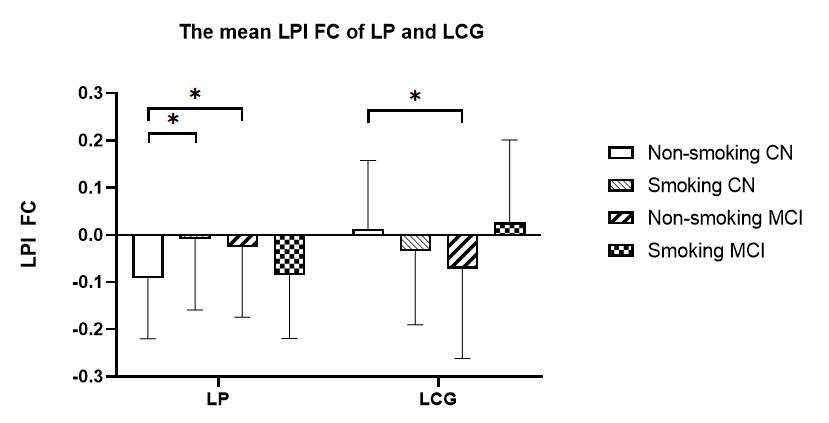


**Figure S2. The mean LPI FC of the LP and LCG of the non-smoking CN, smoking CN, non-smoking MCI, and smoking MCI.**

The figure shows the mean LPI FC of the LP and LCG L of the non-smoking CN, smoking CN, non-smoking MCI, and smoking MCI throughout the 511 subjects. LPI FC with LP in the non-smoking CN was significantly lower than those in the smoking CN and non-smoking MCI (*p* < 0.05, Bonferroni corrected). LPI FC with LCG in the non-smoking CN was significantly higher than in the non-smoking MCI (*p* < 0.05, Bonferroni corrected).

Abbreviation: LPI: left posterior insula, FC: functional connectivity, LP: left precuneus, LCG: left cingulate gyrus, HC: healthy controls, MCI: mild cognitive impairment, * *p* < 0.05, Bonferroni corrected.

## Supplementary Material 4

**Table S2. Demographic characteristics and neuropsychological scales of the 511 subjects**

|  | Non-smoking HC (n=304) | Smoking HC (n=44) | Non-smoking MCI (n=130) | Smoking MCI (n=33) | F/χ^2^ Value | *p*-Value |
| --- | --- | --- | --- | --- | --- | --- |
| Age | 72.98±7.25 | 75.83±7.64 | 74.33±7.87 | 76.61±7.54 | 4.09 | 0.007^b^ |
| Education | 16.88±2.33 | 16.27±92.60 | 16.71±2.54 | 15.79±2.40 | 2.56 | 0.05 |
| Sex (F/M) | 183/121 | 21/23 | 56/74 | 12/21 | 15.62 | 0.001 |
| MMSE | 29.14±1.08 | 29.25±0.81 | 28.33±1.62 | 27.70±1.76 | 22.97 | < 0.001^abcd^ |
| **Visuo-spatial** |  |  |  |  |  |  |
| CDT | 4.76±0.52 | 4.61±0.62 | 4.44±0.91 | 4.49±0.94 | 7.78 | < 0.001^a^ |
| **Memory** |  |  |  |  |  |  |
| IST | 14.64±3.61 | 15.23±3.36 | 10.62±4.30 | 11.64±4.76 | 38.55 | < 0.001^abcd^ |
| DST | 13.53±3.80 | 14.21±3.62 | 8.59±4.37 | 9.61±4.59 | 54.97 | < 0.001^abcd^ |
| AVLT | 46.97±10.10 | 45.11±10.99 | 37.45±10.34 | 37.70±11.37 | 27.12 | < 0.001^abcd^ |
| **Language** |  |  |  |  |  |  |
| SVF | 21.68±5.12 | 21.32±5.87 | 19.02±5.07 | 18.67±5.23 | 10.08 | < 0.001^ab^ |
| **Attention** |  |  |  |  |  |  |
| TMT-A | 31.30±9.51 | 29.84±6.70 | 36.15±13.05 | 37.76±12.91 | 9.96 | < 0.001^abcd^ |
| **Execution** |  |  |  |  |  |  |
| TMT-B | 74.85±34.90 | 70.50±27.24 | 96.23±51.98 | 110.39±64.58 | 14.21 | < 0.001^abcd^ |

Data are presented as mean ± standard deviation. CN: cognitively normal, MCI: mild cognitive impairment, F: female, M: male, MMSE: Mini-Mental State Examination, CDT: clock drawing test, IST: immediate story retell, DST: delayed story retell, AVLT: auditory verbal learning test, SVF: semantic verbal fluency, TMT-A: Trail-Making Test, Part A, TMT-B: Trail-Making Test, Part B.

^a-d^ Post hoc paired comparison further revealed the source of ANOVA difference respectively (*p* < 0.05, Bonferroni corrected) (^a^ non-smoking CN vs. non-smoking MCI, ^b^ non-smoking CN vs. smoking MCI, ^c^ smoking CN vs. non-smoking MCI, ^d^ smoking CN vs. smoking MCI).

**Table S3. Brain areas with significant insula FC difference among non-smoking CN, smoking CN, non-smoking MCI, and smoking MCI in 511 subjects**

| Cluster size | Brain region | Peak MNI coordinates | | | peak-value |
| --- | --- | --- | --- | --- | --- |
|  |  | X | Y | Z |  |
| right anterior insula | |  |  |  |  |
| 216 | right inferior parietal lobule | 51 | -45 | 21 | 28.57 |
| 60 | left middle temporal gyrus | -54 | -51 | 21 | 19.32 |
| left anterior insula | |  |  |  |  |
| 45 | left angular gyrus | -57 | -60 | 27 | 15.12 |
| right posterior insula | |  |  |  |  |
| 21 | right middle occipital gyri | 36 | -90 | 18 | 14.96 |
| left posterior insula | |  |  |  |  |
| 18 | left temporal pole | -21 | 9 | -27 | 18.36 |

Abbreviation: FC: functional connectivity, CN: cognitively normal, MCI: mild cognitive impairment, MNI: Montreal Neurological Institute; X Y Z coordinates the primary peak locations in the MNI space.


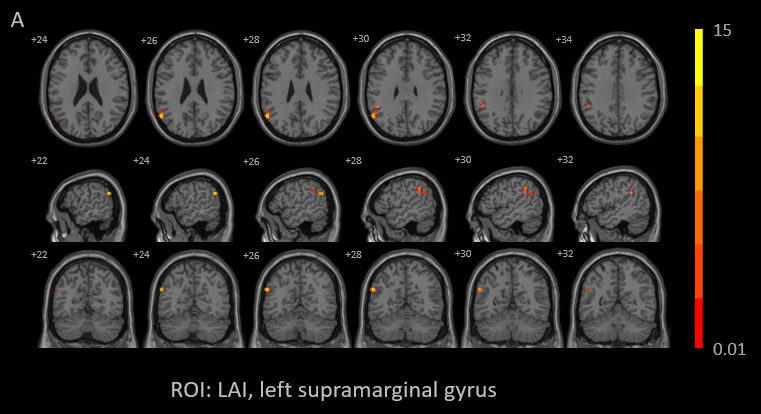


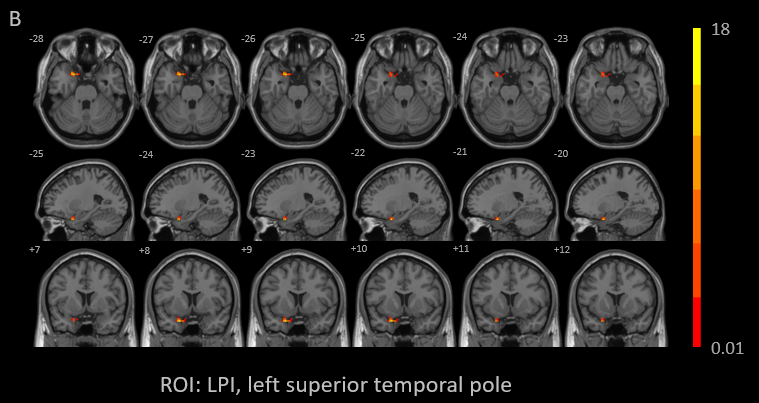


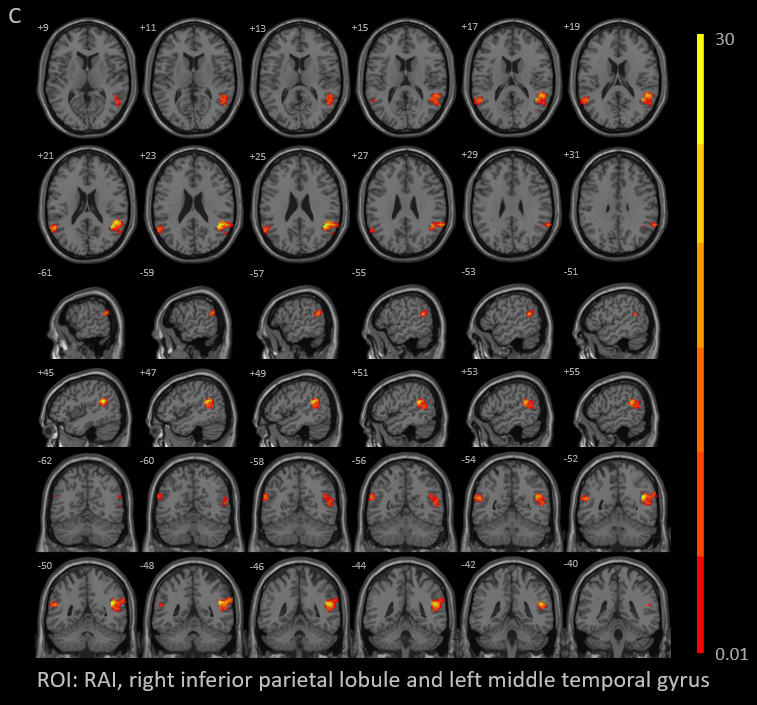


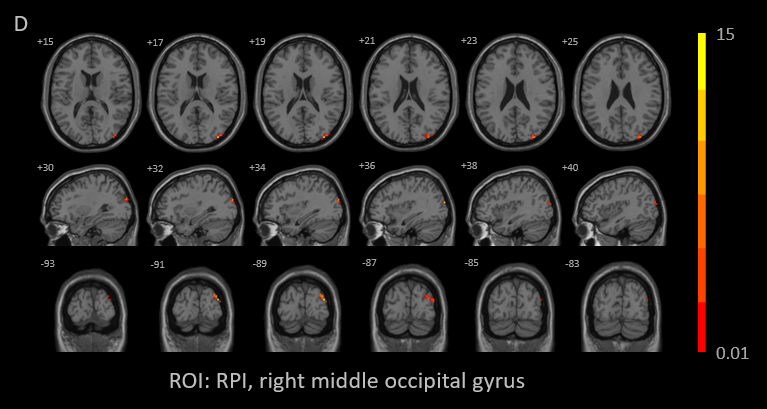


**Figure S3. The significant interaction effects (smoking × cognitive state) identified** **among non-smoking CN, smoking CN, non-smoking MCI, and smoking MCI in 511 subjects.**

Figures A-D respectively shows the difference in the insula FC among non-smoking CN, smoking CN, non-smoking MCI, and smoking MCI in whole subjects. After correction for age, sex, education, and gray matter volume, we identified regions where the FC to the insula were significantly altered: **A)** between the LAI and left supramarginal gyrus (*p* < 0.01, cluster level < 0.05, GRF corrected); **B)** between the LPI and left superior temporal pole (*p* < 0.01, cluster level < 0.05, GRF corrected); **C)** between the RAI and right inferior parietal lobule and left middle temporal gyrus (*p* < 0.01, cluster level < 0.05, GRF corrected); **D)** between the RPI and right middle occipital gyrus (*p* < 0.01, cluster level < 0.05, GRF corrected)

Abbreviation: ROI: region-of-interest, LAI: left anterior insula, LPI: left posterior insula, RAI: right anterior insula, RPI: right posterior insula, CN: cognitively normal, MCI: mild cognitive impairment,


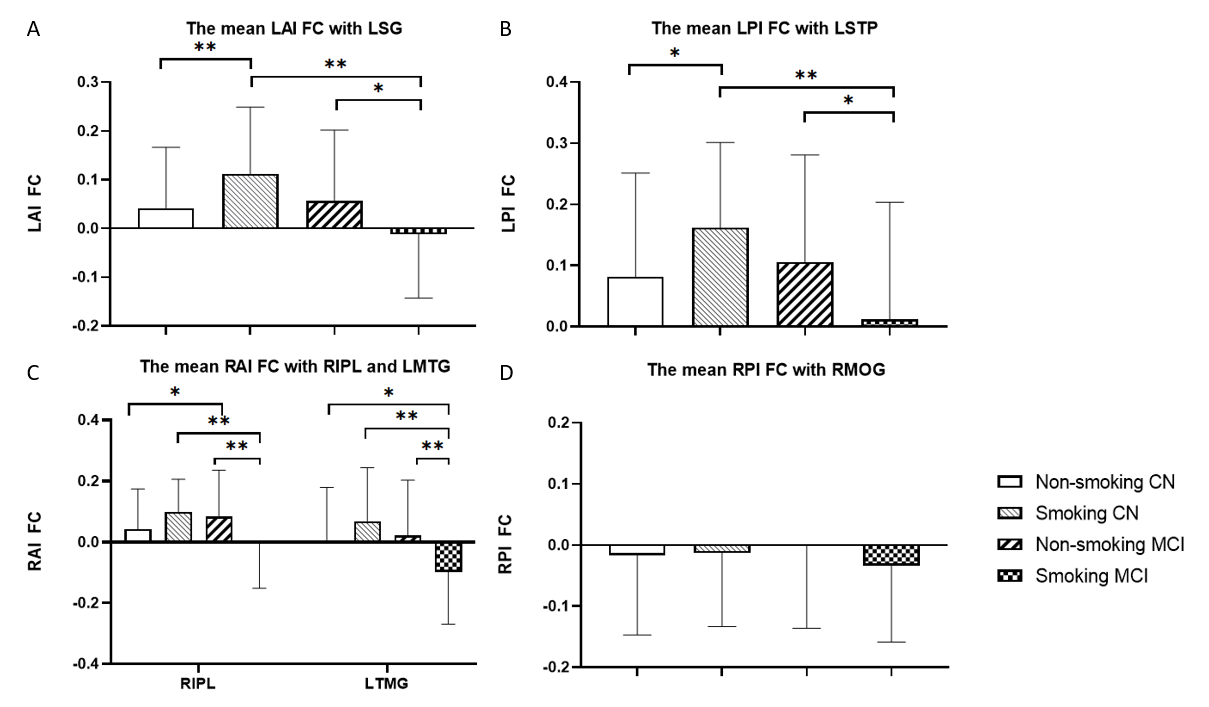


**Figure S4. The mean insula FC of non-smoking CN, smoking CN, non-smoking MCI, and smoking MCI.**

Figures A-D respectively shows the mean insula FC of non-smoking CN, smoking CN, non-smoking MCI, and smoking MCI. **A)** and **B)** LAI-LSG FC and LPI-LSTP FC in the smoking MCI were significantly lower than those in the smoking CN (*p* < 0.01, Bonferroni corrected) and non-smoking MCI (*p* < 0.05, Bonferroni corrected). And insula FC in smoking CN was higher than that in the non-smoking CN both during LAI-LSG (*p* < 0.01, Bonferroni corrected) and LPI-LSTP (*p* < 0.05, Bonferroni corrected). **C)** RAI-RIPL FC in the non-smoking CN was significantly higher than in the non-smoking MCI (*p* < 0.05, Bonferroni corrected). RAI-LMTG in the non-smoking CN was significantly higher than in the smoking MCI (*p* < 0.05, Bonferroni corrected). And RAI-RIPL FC and RAI-LMTG FC in the smoking MCI were significantly lower than those in the smoking CN and non-smoking MCI (*p* < 0.01, Bonferroni corrected).

Abbreviation: LAI: left anterior insula, FC: functional connectivity, LSG: left supramarginal gyrus, LPI: left posterior insula, LSTP: left superior temporal pole, RAI: right anterior insula, RIPL: right inferior parietal lobule, LMTG: left middle temporal gyrus, RPI: right posterior insula, RMOG: right middle occipital gyrus, CN: cognitively normal, MCI: mild cognitive impairment

* *p* < 0.05, Bonferroni corrected, ** *p* < 0.01, Bonferroni corrected

## Supplementary Material 5

**Table S4. Correlations between insula FC and neuropsychological scores in the PSM population**

|  | Total | | Non-smokers | | Smokers | | HC | | MCI | |
| --- | --- | --- | --- | --- | --- | --- | --- | --- | --- | --- |
|  | LMTG | RIPL | LMTG | RIPL | LMTG | RIPL | LMTG | RIPL | LMTG | RIPL |
| MMSE | -0.01 (0.93) | 0.01 (0.84) | -0.08 (0.36) | -0.10 (0.25) | 0.11 (0.36) | 0.28 (0.02) | -0.02 (0.83) | 0.02 (0.82) | -0.09 (0.42) | -0.05 (0.65) |
| **Visuo-spatial** |  |  |  |  |  |  |  |  |  |  |
| CDT | -0.001 (0.99) | -0.11 (0.13) | -0.03 (0.75) | -0.13 (0.12) | 0.02 (0.86) | -0.09 (0.48) | -0.04 (0.69) | -0.11 (0.23) | 0.01 (0.97) | -0.14 (0.20) |
| **Memory** |  |  |  |  |  |  |  |  |  |  |
| IST | -0.01 (0.91) | -0.07 (0.34) | -0.15 (0.09) | -0.21 (0.02) | 0.23 (0.05) | 0.23 (0.05) | 0.05 (0.56) | -0.06 (0.48) | -0.20 (0.07) | -0.15 (0.18) |
| DST | -0.04 (0.57) | -0.11 (0.11) | -0.19 (0.03) | **-0.31 (<0.001^)^** | 0.20 (0.09) | 0.30 (0.01) | -0.01 (0.94) | -0.07 (0.42) | -0.25 (0.03) | -0.27 (0.02) |
| AVLT | 0.05 (0.48) | -0.01 (0.88) | -0.03 (0.77) | -0.13 (0.13) | 0.17 (0.17) | 0.25 (0.03) | 0.15 (0.10) | -0.01 (0.89) | -0.22 (0.05) | -0.11 (0.33) |
| **Language** |  |  |  |  |  |  |  |  |  |  |
| SVF | 0.06 (0.41) | 0.004 (0.95) | -0.07 (0.44) | -0.12 (0.18) | 0.23 (0.05) | 0.26 (0.03) | 0.08 (0.36) | -0.01 (0.91) | -0.05 (0.67) | -0.02 (0.88) |
| **Attention** |  |  |  |  |  |  |  |  |  |  |
| TMT-A | -0.07 (0.29) | -0.10 (0.14) | -0.02 (0.81) | 0.02 (0.82) | -0.16 (0.17) | **-0.35 (0.003^)^** | -0.06 (0.50) | -0.13 (0.14) | -0.02 (0.86) | -0.05 (0.64) |
| **Execution** |  |  |  |  |  |  |  |  |  |  |
| TMT-B | -0.12 (0.10) | -0.11 (0.13) | -0.04 (0.64) | 0.09 (0.32) | -0.22 (0.06) | **-0.45 (<0.001^)^** | -0.08 (0.37) | 0.004 (0.97) | -0.09 (0.42) | -0.17 (0.13) |

Abbreviation: FC: functional connectivity, CN: cognitively normal, MCI: mild cognitive impairment, MMSE: Mini-Mental State Examination, CDT: clock drawing test, IST: immediate story retell, DST: delayed story retell, AVLT: auditory verbal learning test, SVF: semantic verbal fluency, TMT-A: Trail-Making Test, Part A, TMT-B: Trail-Making Test, Part B.

^a^ *p* < 0.05/16, Bonferroni corrected


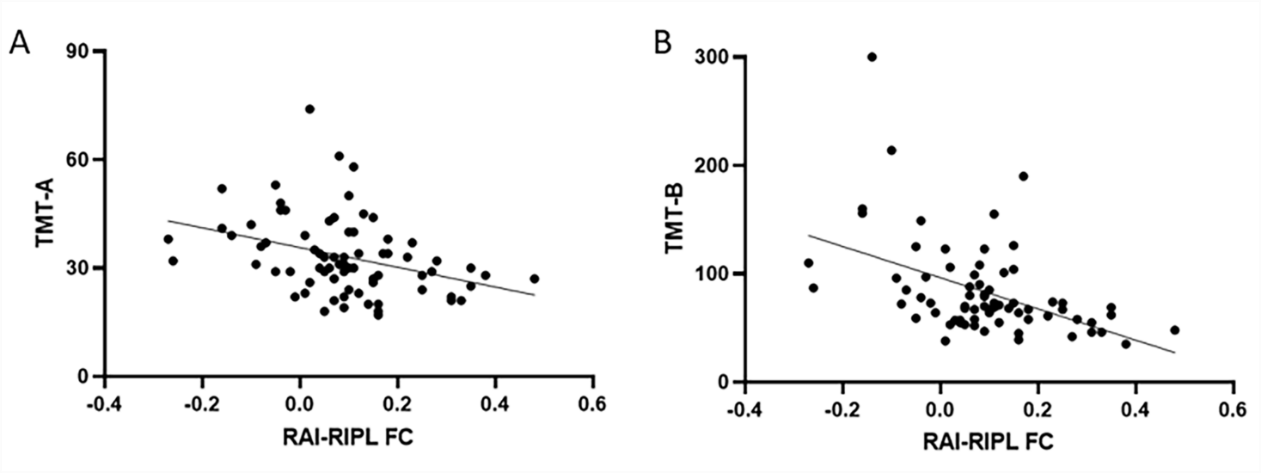


**Figure S5. Correlation between insula FC and neuropsychological scores in smokers in the PSM population.**

Figures A and B respectively represent the correlation between RAI-RIPL FC and TMT-A (r = -0.35, p < 0.05/16) and the correlation between RAI-PIPL FC with TMT-B (r = -0.45, p < 0.05/16) after correction for age, sex and education among the smoking groups.

Abbreviation: FC: functional connectivity, PSM: propensity score matching, RAI: right anterior insula, RIPL: right inferior parietal lobule, DST: delayed story retell, TMT-A: Trail-Making Test, Part A, TMT-B: Trail-Making Test, Part B.
